# Supplementary material for: Causal Relationship between Aging and Anorexia Nervosa: A White-Matter-Microstructure-Mediated Mendelian Randomization Analysis
Source: Biomedicines. 2024 Aug 16;12(8):1874. doi: 10.3390/biomedicines12081874 (PMC11351342; doi:10.3390/biomedicines12081874)
Supplement: Supplementary file 1 [file biomedicines-12-01874-s001.zip › Supplementary Figure/Figure S4/Singe SNP plot of MR analysis for TL to ubm-b-1465.pdf]

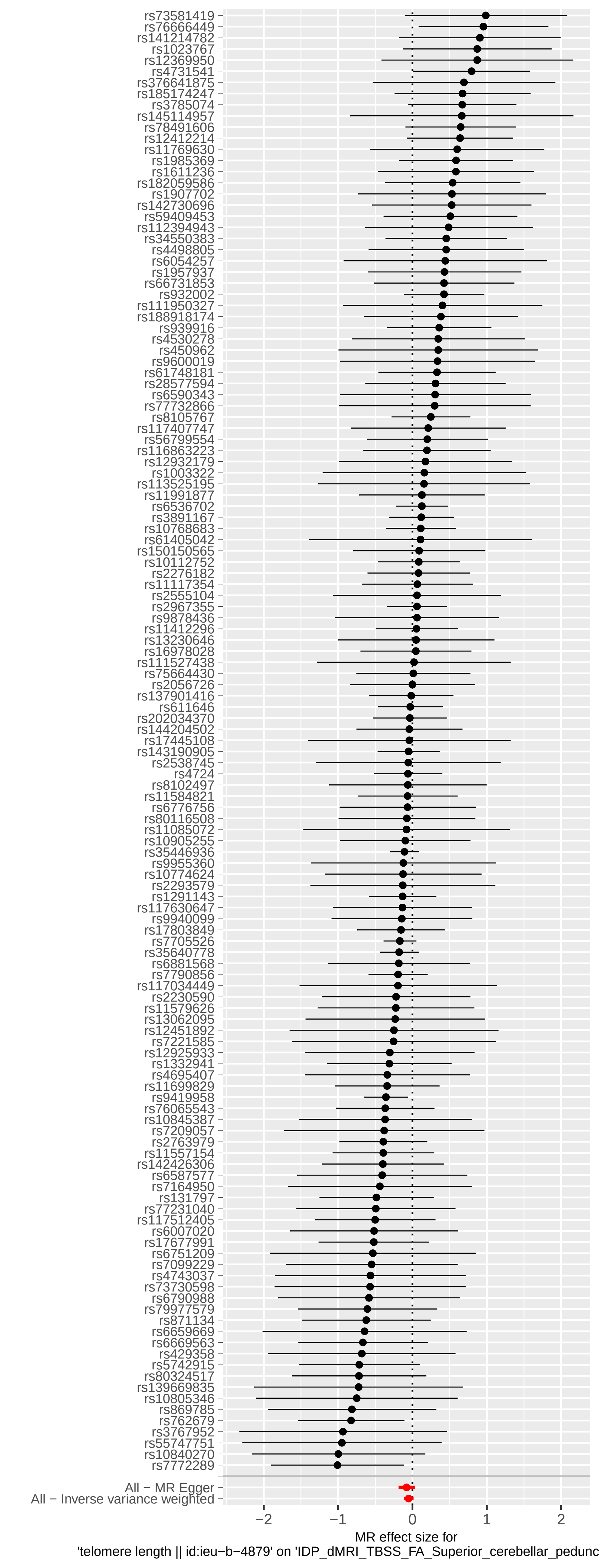

rs73581419
rs76666449
rs141214782
rs1023767
rs12369950
rs4731541
rs376641875
rs185174247
rs3785074
rs145114957
rs78491606
rs12412214
rs11769630
rs1985369
rs1611236
rs182059586
rs1907702
rs142730696
rs59409453
rs112394943
rs34550383
rs4498805
rs6054257
rs1957937
rs66731853
rs932002
rs111950327
rs188918174
rs939916
rs4530278
rs450962
rs9600019
rs61748181
rs28577594
rs6590343
rs77732866
rs8105767
rs117407747
rs56799554
rs116863223
rs12932179
rs1003322
rs113525195
rs11991877
rs6536702
rs3891167
rs10768683
rs61405042
rs150150565
rs10112752
rs2276182
rs11117354
rs2555104
rs2967355
rs9878436
rs11412296
rs13230646
rs16978028
rs111527438
rs75664430
rs2056726
rs137901416
rs611646
rs202034370
rs144204502
rs17445108
rs143190905
rs2538745
rs4724
rs8102497
rs11584821
rs6776756
rs80116508
rs11085072
rs10905255
rs35446936
rs9955360
rs10774624
rs2293579
rs1291143
rs117630647
rs9940099
rs17803849
rs7705526
rs35640778
rs6881568
rs7790856
rs117034449
rs2230590
rs11579626
rs13062095
rs12451892
rs7221585
rs12925933
rs1332941
rs4695407
rs11699829
rs9419958
rs76065543
rs10845387
rs7209057
rs2763979
rs11557154
rs142426306
rs6587577
rs7164950
rs131797
rs77231040
rs117512405
rs6007020
rs17677991
rs6751209
rs7099229
rs4743037
rs73730598
rs6790988
rs79977579
rs871134
rs6659669
rs6669563
rs429358
rs5742915
rs80324517
rs139669835
rs10805346
rs869785
rs762679
rs3767952
rs55747751
rs10840270
rs7772289
All − MR Egger
All − Inverse variance weighted
−2
−1
0
1
2
MR effect size for
'telomere length || id:ieu−b−4879' on 'IDP_dMRI_TBSS_FA_Superior_cerebellar_pedunc
